# Cognate antigen engagement on parenchymal cells stimulates CD8^+^ T cell proliferation *in situ*

**DOI:** 10.1038/ncomms14809

**Published:** 2017-04-12

**Authors:** Robyn M. Sutherland, Sarah L. Londrigan, Jamie L. Brady, Emma M. Carrington, Julia M. Marchingo, Susanne Heinzel, Philip D. Hodgkin, Kate L. Graham, Thomas W. Kay, Yifan Zhan, Andrew M. Lew

**Affiliations:** 1 Immunology Division, The Walter and Eliza Hall Institute of Medical Research, Parkville, Victoria 3052, Australia; 2 Department of Medical Biology, The University of Melbourne, Parkville, Victoria 3010, Australia; 3 Department of Microbiology and Immunology, The University of Melbourne, at the Peter Doherty Institute for Infection and Immunity, Melbourne, Victoria 3000, Australia; 4 St Vincent's Institute, Fitzroy, Victoria 3065, Australia; 5 Department of Medicine, St Vincent's Hospital, The University of Melbourne, Fitzroy, Victoria 3065, Australia

## Abstract

T-cell responses are initiated upon cognate presentation by professional antigen presenting cells in lymphoid tissue. T cells then migrate to inflamed tissues, but further T-cell stimulation in these parenchymal target sites is not well understood. Here we show that T-cell expansion within inflamed tissues is a distinct phase that is neither a classical primary nor classical secondary response. This response, which we term ‘the mezzanine response', commences within days after initial antigen encounter, unlike the secondary response that usually occurs weeks after priming. A further distinction of this response is that T-cell proliferation is driven by parenchymal cell antigen presentation, without requiring professional antigen presenting cells, but with increased dependence on IL-2. The mezzanine response might, therefore, be a new target for inhibiting T-cell responses in allograft rejection and autoimmunity or for enhancing T-cell responses in the context of microbial or tumour immunity.

## Abstract (web summary)

Professional antigen presenting cells (APC) are the major activator of T cells that then hone to sites of inflammation. Using islet cell grafts, here the authors show that parenchymal cells can present antigen to activate CD8^+^ T cells at inflammatory sites, coining this a ‘mezzanine response' distinct from primary and secondary responses associated with professional APCs.

T-cell immune responses have a central function in both the resolution of infectious disease and the pathology of organ-specific autoimmunity and transplant rejection. Specificity of the response is imbued by cognate interaction of clonal T-cell receptors (TCR) with peptide antigen presented on MHC class I (in case of CD8^+^ T cells) or MHC class II (in case of CD4^+^ T cells) molecules. These responses are initiated within lymphoid tissues when T cells encounter cognate antigen presented on professional antigen presenting cells (APC), most notably dendritic cells (DC)^1^. Early studies showed that primary immune responses require the antigen to enter secondary lymphoid organs, for example via afferent lymph^2^,^3^. The requirement of secondary lymphoid organs has been shown more elegantly in mice without spleens and lymph nodes (LN), which no longer respond to viral challenge^4^. Moreover, the requirement of cognate MHC on haematopoietic cells (and therefore not parenchymal cells) has also been shown^5^,^6^. The initial findings that DCs are key to stimulating primary mixed lymphocyte reactions^7^ were followed up by *in vivo* findings that depletion of DCs abrogates cytotoxic T-cell responses to Listeria and Plasmodium^8^.

Subsequent to antigen recognition, T-cell proliferation and differentiation can be detected within the lymphoid tissue, with the conventional implication that the magnitude and type of T-cell response is controlled by the lymphoid site. The parenchyma within inflamed tissues (for example, virally infected epithelia) is generally viewed as passive targets of T cells that have been primed in lymphoid tissues. Indeed, ongoing recruitment of T cells has been shown to be one contributor to tissue inflammation^9^. However, some studies have suggested that there is further expansion within inflamed tissues driven by local antigen presentation. Local antigen presentation is able to promote entry into non-lymphoid tissues^10^,^11^,^12^ as well as local proliferation^13^,^14^ and survival^15^,^16^ of infiltrating T cells, thus refining the potency and duration of the response. However, the APCs that drive the local immune response are incompletely defined. Some studies report that professional APCs, namely DC, are important to local T-cell expansion via promotion of T-cell recruitment, proliferation and survival^12^,^13^,^15^,^16^. Cognate interactions with non-professional stromal APC, particularly endothelial cells of the blood vessels, are also thought to contribute to T-cell recruitment^10^,^11^. The role of cognate interactions with parenchymal cells is less clear.

Parenchymal cells present antigen on MHC class I during infection, autoimmunity and allogeneic responses as targets for CD8^+^ T-cell killing. In this study, we investigate the role of parenchymal cells as APCs, not merely as targets, but in the promotion of immune responses. We develop an *in vivo* model that enables us to define the function of parenchymal cell antigen presentation in expansion of the CD8^+^ T-cell response in inflamed tissues.

## Results

### Cognate CD8^+^ T cells are found in LN and inflamed tissue

**Figure 1:**
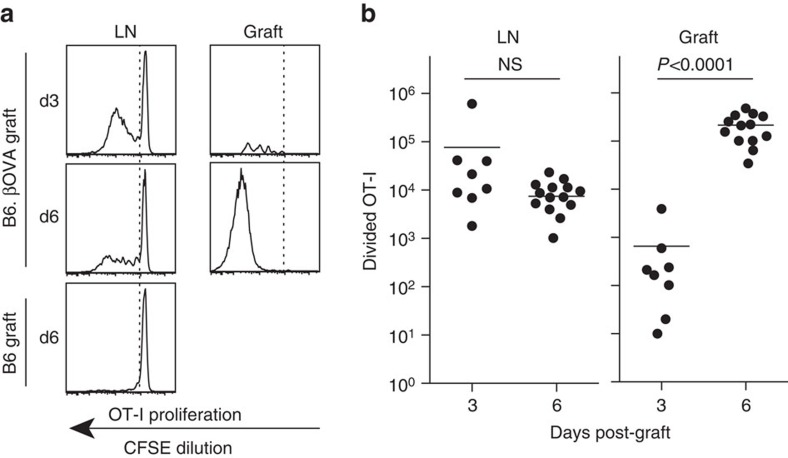
CD8^+^ T cells are primed in draining LN then expand at the site of inflammation. Divided OT-I cells (viable CD45.1^+^CD8^+^Vα2^+^ gate) in draining renal LN and graft 3 or 6 days after receipt of a single graft of 400 B6.βOVA islets. (**a**) Representative flow cytometry plots. Position of the undivided OT-I peak was determined using ‘no antigen' control of a B6 islet graft. (**b**) Total number of divided OT-I in renal LN and graft where each point represents an individual mouse. Pooled data from seven independent experiments: *n*=8 graft recipients at day 3 and *n*=14 graft recipients at day 6. One day 6 graft was lost due to a flow cytometer malfunction. Horizontal bars are means, *P* values were calculated by unpaired, two-tailed *t*-test with Welch's correction.

In initial experiments (Fig. 1) we established a model in which CD8 T-cell responses to a parenchymal antigen, ovalbumin (OVA), could be analysed. OT-I mice express a transgenic TCR that mediates CD8^+^ T-cell recognition of OVA_257-264_ peptide presented on the MHC class I molecule H-2K^b^. CD8 T cells were enriched from OT-I/CD45.1 mice, CFSE dye-labelled and adoptively transferred by i.v. into B6 (CD45.2^+^) host mice. Adoptively transferred T cells could be distinguished from host cells by CD45.1 staining (as well as CD8 and the Vα2 chain of the OT-I TCR) while CFSE-dye dilution was used to identify cells that had proliferated (for example, Fig. 1a, Supplementary Fig. 1). Parenchymal antigen was introduced by grafting B6.βOVA islets (such islets express OVA in parenchymal β cells under the rat insulin promoter, Supplementary Table 1) beneath the renal capsule of host mice that had already received OT-I/CD45.1 T cells. All three sources (host, T-cell donor and islet donor) have a B6 background and hence express H-2K^b^. Thus, although OVA antigen expression was limited to grafted β cells, we assumed that presentation of β-cell-derived OVA by non-parenchymal APC such as DC via cross-presentation would be required for LN priming and perhaps local T-cell responses^17^,^18^.

### CD8^+^ T cells expand at the site of inflammation

As expected, a potent OT-I response was detected first in the renal LN that drains the graft site and this was dependent on the presence of OVA antigen (Fig. 1a,b). At 3 days post-graft, divided OT-I could be detected in LNs draining the B6.βOVA islet grafts (Fig. 1a) and were abundant (Fig. 1b, mean=9.2 × 10^4^). By contrast, 100-fold fewer divided OT-I cells could be detected within B6.βOVA grafts (Fig. 1a,b, mean=6.6 × 10^2^). The number of divided OT-I cells increased 300-fold at the graft site from day 3 to day 6 (Fig. 1b, mean=6.6 × 10^2^ at day 3 to 2.1 × 10^5^ at day 6, *P*<0.0001, unpaired two-tailed *t*-test with Welch's correction) whereas there was no increase in the LN (Fig. 1b). The OT-I response in B6.βOVA grafts could be explained by infiltration of OT-I cells that had proliferated in the LN. However, CFSE profiles were different between the graft and LN sites as OT-I cells in grafts were more highly divided than those in the LN at day 6 (Fig. 1a). There are at least two possible explanations for this difference. Only cells that had divided many times infiltrated the graft. Alternatively, the cells reaching the graft more rapidly divided, possibly due to the abundance of available antigen. The increase in absolute cell numbers in the graft compared with LN at day 6 led us to investigate this latter explanation.

### Cognate interaction with islets promotes expansion

**Figure 2:**
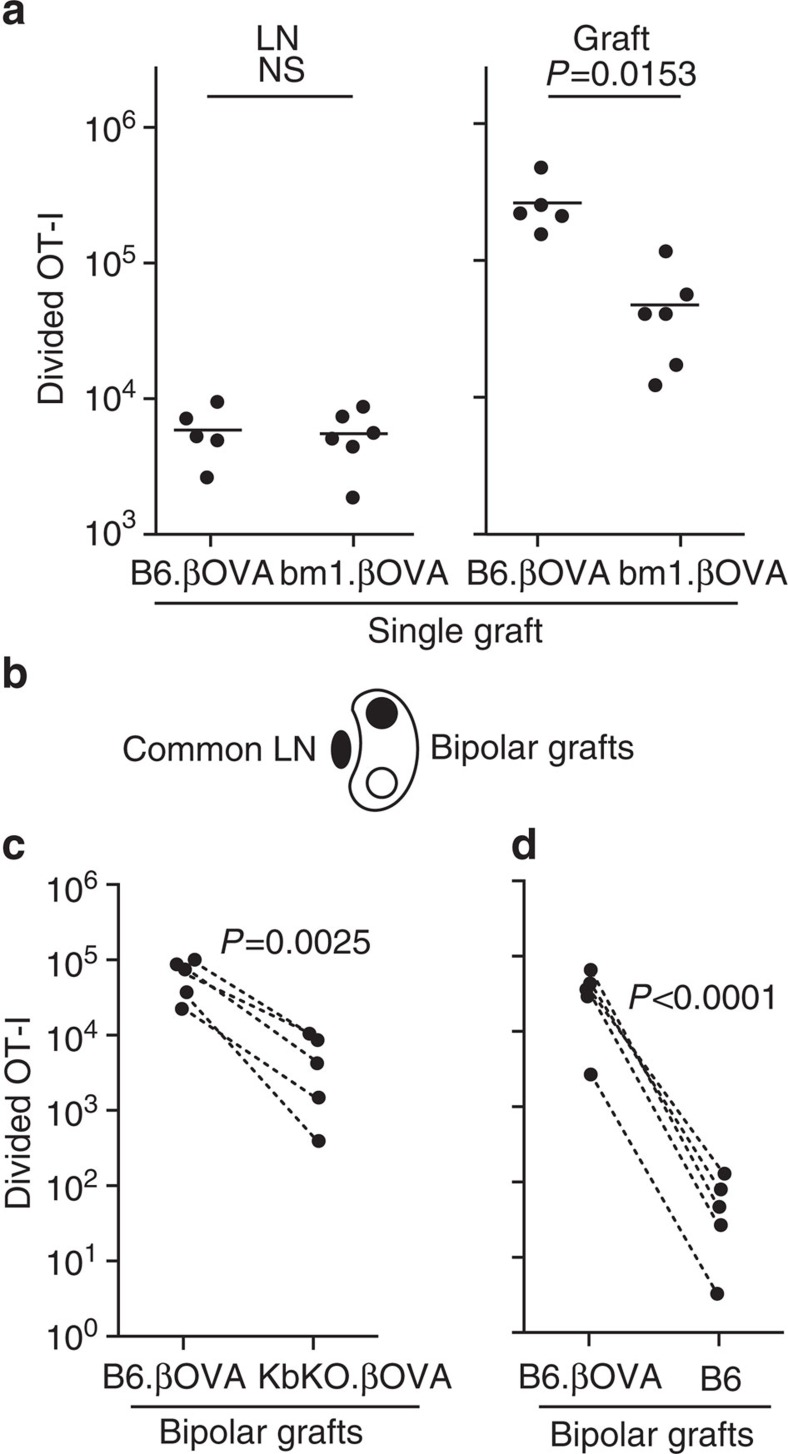
Cognate interaction with islet cells contributes to CD8^+^ T-cell expansion at the site of inflammation. Flow cytometry analysis of OT-I cells (viable CD45.1^+^CD8^+^Vα2^+^ gate) 6 days post graft. (**a**) Total divided OT-I in the draining renal LN and graft after receipt of a single graft of 400 B6.βOVA or bm1.βOVA islets. Data for B6.βOVA grafted mice are a subset of that shown in Fig. 1b. Each point represents an individual mouse. Pooled data from two independent experiments are shown in each panel: *n*=5 recipients of B6.βOVA grafts, and *n*=6 recipients of bm1.βOVA grafts. Horizontal bars are means, *P* values were calculated by unpaired, two-tailed *t*-test with Welch's correction. (**b**) Schematic of bipolar graft model in which grafts of 200 islets are placed at opposite poles of the same kidney and share a common draining renal LN. Total divided OT-I in (**c**) B6.βOVA and KbKO.βOVA bipolar grafts and (**d**) B6.βOVA and B6 bipolar grafts (*n*=5 recipient mice pooled from two independent experiments in each of **c** and **d**) Data for the same mouse are connected by dashed lines: *P* values were calculated by ratio paired *t*-test.

To determine the possible contribution of cognate MHC in the graft to local expansion we used islets derived from bm1.βOVA mice (Supplementary Table 1). In bm1.βOVA mice, the H-2K^bm1^ molecule is unable to present the OVA_257–264_ peptide due to three mutations to H-2K^b^ (ref. 19). A comparison of responses to B6.βOVA and bm1.βOVA islet grafts showed that despite similar OT-I responses in the draining renal LN, expansion of OT-I was reduced sixfold in bm1.βOVA compared to B6.βOVA grafts (mean=4.7 × 10^4^ compared to 2.6 × 10^5^ divided OT-I; *P*=0.0153 unpaired two-tailed *t*-test with Welch's correction; Fig. 2a). This less efficient expansion of OT-I cells infiltrating bm1.βOVA grafts suggested that cognate interactions with grafted islet cells contributed to local T-cell expansion.

To eliminate any contribution by H-2K^bm1^ alloantigen we generated KbKO.βOVA mice that express OVA antigen in islet beta cells but lack alloantigen or H-2K^b^ (Supplementary Table 1). We then used a ‘bipolar graft model' to compare responses to B6.βOVA and KbKO.βOVA islet grafts placed at opposite poles of the same kidney in a B6 host mouse such that graft-infiltrating T cells were derived from T cells primed in the same LN (Fig. 2b). There were 10–100-fold more T cells present in B6.βOVA than KbKO.βOVA grafts (Fig. 2c). Using the ratio of divided OT-I at opposite poles of the kidney calculated for individual mice, we derived a Relative Response Ratio (RRR). The RRR indicated that local T-cell expansion was 26-fold greater when cognate engagement with graft cells occurred (RRR=26.4±14.1 in B6.βOVA:KbKO.βOVA grafts, *P*=0.0025, ratio paired *t*-test, Fig. 2c).

We also compared the responses to bipolar B6.βOVA and B6 (no antigen) grafts (Fig. 2d). Very few OT-I could be detected within B6 grafts (Fig. 2d) and we consider it likely that many of these were circulating OT-I derived from blood contamination. Thus, non-specific inflammation associated with transplantation of grafts that lack antigen does not lead to efficient T-cell expansion within the inflamed tissue. By contrast, the response to B6.βOVA grafts was again very potent and more than 900-fold greater than that of the B6 grafts (RRR=950±252 for B6.βOVA:B6 grafts, *P*<0.0001, ratio paired *t*-test, Fig. 2d).

Together these experiments indicated that CD8 T-cell expansion within the inflamed target tissue was significantly promoted by local antigen presentation. This does not necessarily exclude other contributions to T-cell expansion, for example, by cross-presenting host DC in the graft. We only aver that cognate interaction between T cells and islet cells results in substantial T-cell expansion.

### Cognate interaction with resident leukocytes is not required

**Figure 3:**
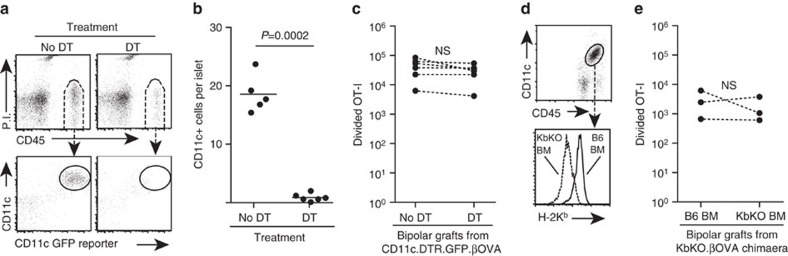
Cognate interaction with IRL does not significantly contribute to CD8^+^ T-cell expansion. Flow cytometry analysis showing depletion of CD11c^+^ IRL in CD11c.DTR.GFP mice treated with DT: (**a**) Representative plots showing gating of CD11c^+^IRL in islets from untreated control mice and their depletion on the day following a single DT treatment, and (**b**) enumeration of CD11c^+^IRL in untreated and DT treated mice. Each point represents an individual islet preparation containing pooled islets from 1 to 8 mice, *n*=5 untreated islet preparations and 6 DT treated islet preparations pooled from four independent experiments. *P* value calculated by unpaired, two-tailed *t*-test with Welch's correction. (**c**) Total divided OT-I at 6 days post bipolar graft of islets obtained from CD11c.DTR.GFP.βOVA mice that were untreated or treated with DT (*n*=6 recipient mice pooled from two independent experiments). Data for the same mouse are connected by dashed lines; *P* values calculated by ratio paired *t*-test. (**d**) Representative plots showing absence or presence of H-2K^b^ expression on CD11c^+^IRL of KbKO mice reconstituted with KbKO or B6 BM respectively. Pregated on viable cells. (**e**) Total divided OT-I at 6 days post bipolar graft of islets obtained from chimaeric mice (*n*=3 recipient mice from a single experiment). Data for the same mouse are connected by dashed lines; *P* values calculated by ratio paired *t*-test.

Islets are complex clusters of cells that include β cells, endothelial cells and islet resident leukocytes (IRL). Therefore, we next sought to clarify which islet cells presented antigen to infiltrating T cells to drive T-cell expansion. The most obvious candidate would be IRL. These CD45^+^ cells have variously been defined as DC or macrophages, co-express CD11c and F4/80, and have been shown to process and present antigens^20^,^21^,^22^,^23^. We therefore examined the role of donor IRL in driving local CD8 T-cell expansion. B6.βOVA mice were crossed to CD11c.DTR.GFP mice in order to obtain CD11c.DTR.GFP.βOVA mice whose β cells express OVA and whose CD11c^+^ cells could be ablated by diphtheria toxin (DT) injection^8^. We first tested by FACS that islets isolated from CD11c.DTR.GFP mice treated with DT were depleted of CD11c^+^ cells (Fig. 3a,b). CD11c^+^ IRL in untreated control mice were identified by staining for CD45 and CD11c as well as their expression of the GFP reporter and were clearly depleted by DT treatment (Fig. 3a,b, *P*=0.0002, unpaired two-tailed *t*-test with Welch's correction). We also confirmed that the CD11c^+^IRL co-expressed F4/80 and H-2 Kb MHC class I (Supplementary Fig. 2a) and consequently F4/80^+^ cells were also depleted from the islets of DT treated mice (Supplementary Fig. 2b). CD11c.DTR.GFP.βOVA islets isolated from untreated and DT treated mice were grafted at opposite poles of the same kidney. Divided OT-I number was similar between the grafts, indicating that IRL depletion had no effect on CD8^+^ T-cell expansion at the graft site (Fig. 3c). To confirm that IRL were not important for local CD8 T-cell expansion, we performed a second set of experiments. KbKO.βOVA mice were irradiated and reconstituted with either KbKO or B6 bone marrow (BM) to generate a source of βOVA islets in which H-2Kb expression was absent from all cells or selectively restored to IRL (Fig. 3d). Comparison of such islets in the bipolar graft model showed similar levels of OT-I cell expansion in both grafts (Fig. 3e). Hence, IRL are not major drivers of local CD8 T-cell expansion.

### Cognate interaction with parenchymal cells is important

**Figure 4:**
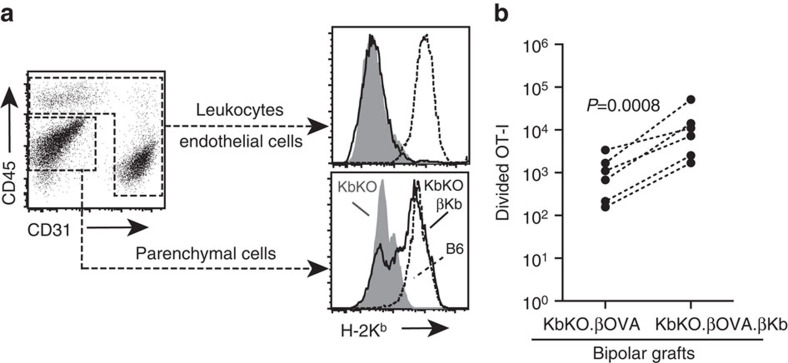
Cognate interaction with islet parenchymal β cells contributes to CD8^+^ T-cell expansion. (**a**) Flow cytometry plots (gated on viable cells) showing selective restoration of H-2K^b^ expression on parenchymal cells. Histograms represent islets from KbKO (shaded), B6 (dashed) or KbKO.βKb (bold) mice. (**b**) Total divided OT-I at 6 days post bipolar graft of KbKO.βOVA and KbKO. βOVA.βKb. islets (*n*=6 recipient mice pooled from three independent experiments). Data for the same mouse are connected by dashed lines; *P* values calculated by ratio paired *t*-test.

B6.RIP-Kb mice transgenically express H-2K^b^ in β cells under the control of the rat insulin promoter^24^. By crossing these mice to KbKO.βOVA mice we generated KbKO.βOVA.βKb mice in which H-2K^b^ expression was limited to β cells (Supplementary Table 1). Expression of H-2K^b^ on β cells was confirmed by FACS analysis of islets (Fig. 4a). We gated on islet hematopoietic cells (CD45^+^) and endothelial cells (CD31^+^) and showed that H-2K^b^ was not expressed on these cells in either KbKO.βOVA or KbKO.βOVA.βKb mice compared to strong expression in B6 mice (Fig. 4a). The remaining CD45^−^CD31^−^ cells are autofluorescent positive and enriched in endocrine cells. FACS analysis of islets from KbKO.βOVA.βKb mice showed H-2K^b^ expression within the β-cell-enriched autofluorescent population at levels similar to that seen in B6 islets (Fig. 4a). Comparison in the bipolar graft model indicated that expansion of OT-I was 14-fold greater when cognate antigen was expressed on β cells (RRR=14.0±4.1 for KbKO.βOVA.βKb: KbKO.βOVA grafts, *P*=0.0008, ratio paired *t*-test, Fig. 4b).

### T cells proliferate at the site of inflammation

**Figure 5:**
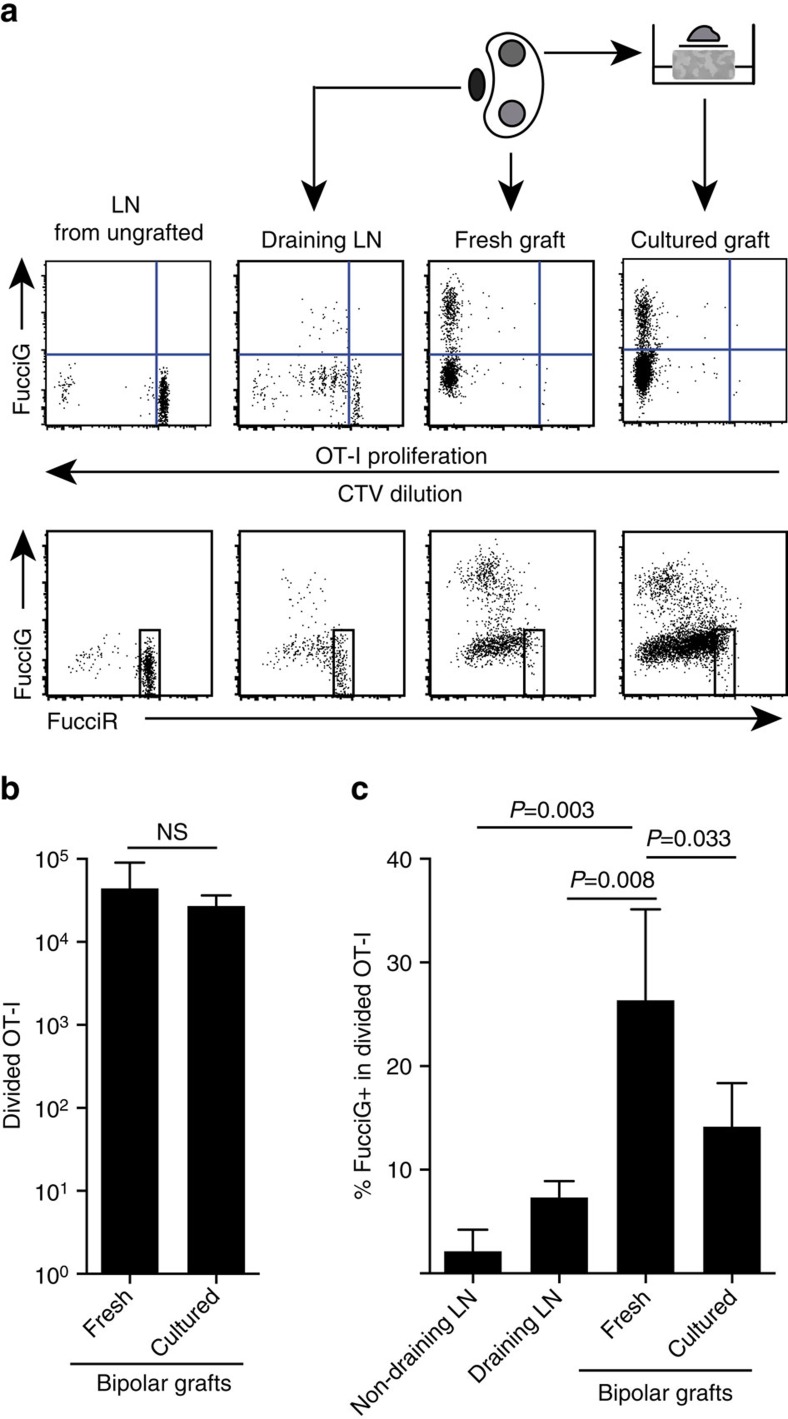
CD8^+^ T-cell proliferation at the site of inflammation contributes to T-cell expansion. (**a**) Schematic and representative flow cytometry plots (gated on viable CD45.1^+^CD8^+^Vα2^+^ lymphocytes) showing response by FucciOT-I cells in renal LNs or bipolar B6.βOVA grafts. LNs and the graft from one pole were examined immediately (fresh at 6 days post graft) while the graft from the opposite pole was cultured for 1 day before analysis. LN from an ungrafted mouse was included to show absence of division and FucciG expression (upper panel) and predominance of FucciR high cells (lower panel) in quiescent FucciOT-I. (**b**) Total divided FucciOT-I in fresh and cultured grafts. Mean+s.d., *n*=5, *P* values calculated by two-tailed ratio paired *t*-test. (**c**) %FucciG^+^ divided OT-I in fresh non-draining and graft draining renal LN as well as fresh and cultured grafts. Mean+s.d., *n*=5 with exception of non-draining LN for which *n*=4 due to loss of one LN during processing. *P* values were calculated by two-tailed unpaired *t*-test with Welch's correction. Data are pooled from two independent experiments.

Our above findings indicate that there was local proliferation at the target site. In order to detect actively proliferating cells we crossed OT-I /CD45.1 mice to FucciRG mice to generate FucciRG/OT-I/CD45.1 mice (abbreviated FucciOT-I). In such mice, cells fluoresce red (FucciR) during G0/G1 and green (FucciG) during S/G2/M cell cycle phases^25^,^26^. The gradual degradation and accumulation of FucciR and FucciG reporters during transition between cycle phases enables further distinctions to be made: more intense FucciR expression in quiescent G0 versus cycling G1 cells, double negative FucciR^−^G^−^ cells in very early G1, and weakly double positive FucciR^+^G^+^ cells in G1/S (refs 26, 27). Quiescent FucciOT-I cells from ungrafted mice were FucciG^−^ (Fig. 5a, upper panel, ungrafted). To avoid interference with the Fucci dyes we replaced CFSE with CTV. In mice that had received B6.βOVA grafts, analysis of CTV dilution indicated that divided FucciOT-I were present in the draining renal LN and some of these were FucciG^+^ suggestive of active cell division (Fig. 5a, upper panel, draining LN). At the graft site, we detected FucciOT-I that had undergone many divisions (CTV no longer detectable) and many of these were FucciG^+^ supportive of ongoing proliferation (Fig. 5a, upper panel, fresh graft). Comparison of the proportion of FucciG^+^ cells within the divided OT-I population (Fig. 5c) indicated that ongoing proliferation within fresh grafts (26.8±8.8% FucciG^+^) exceeded that in either the draining renal LN (7.3±1.6%, *P*=0.008, unpaired two-tailed *t*-test with Welch's correction) or non-draining renal LN (2.1±2.1%, *P*=0.003, unpaired two-tailed *t*-test with Welch's correction). Indeed, examination of both FucciG and FucciR expression indicated that most graft-infiltrating OT-I were actively dividing, that is, very few cells exhibited high expression of FucciR that was a characteristic of quiescent cells in the LN of ungrafted mice (Fig. 5a, lower panels). Although we surmised that this proliferation at the site of inflammation was generated *in situ*, we could not completely discount the possibility that this simply reflected recent arrival of proliferating cells. To clarify this, we harvested the bipolar grafts of B6.βOVA islets to analyse one graft immediately after excision, and culture the other graft for 1 day (Fig. 5a). While the number of OT-I did not increase over the culture period (Fig. 5b), presumably reflecting cell death under suboptimal *ex-vivo* conditions, many FucciG^+^ OT-I cells continued to be detected (14.2±4.2% FucciG^+^, Fig. 5c). The profile of the cultured graft, without possible recruitment for a day, would indicate that there was high *de novo* proliferation in the graft.

### Cognate interaction with parenchymal cells drives proliferation

**Figure 6:**
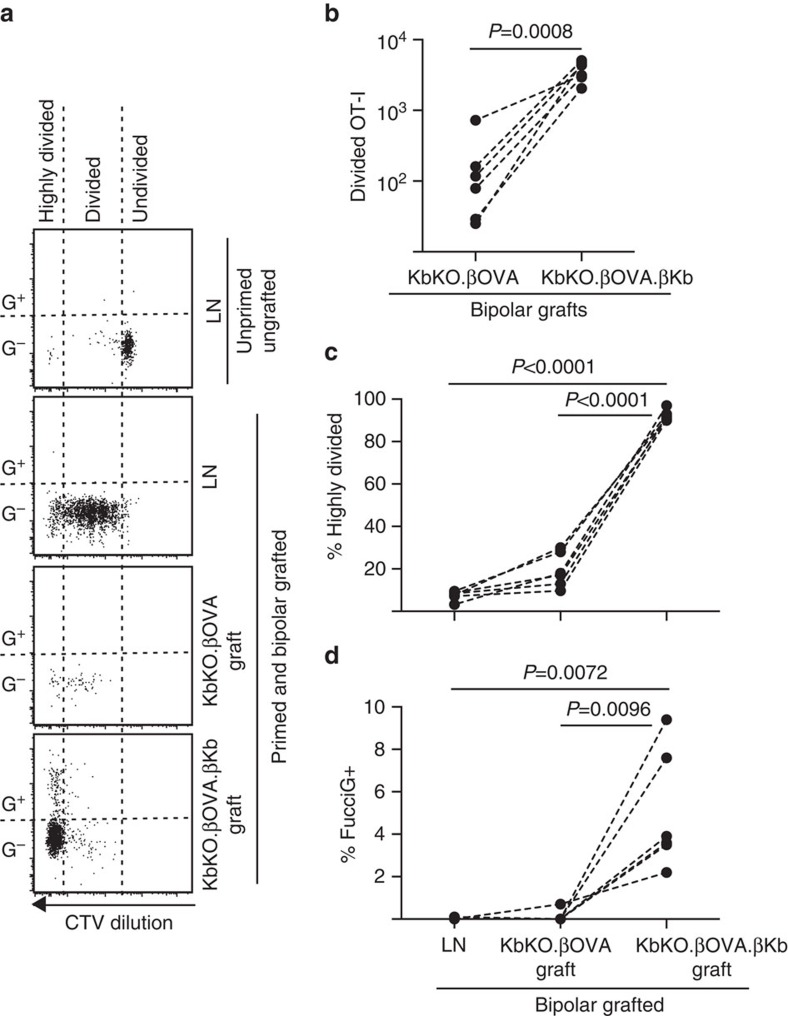
Cognate interaction with islet parenchymal β cells drives CD8^+^ T-cell proliferation. FucciOT-I response to grafts in KbKO BM into B6 host mice in which host hematopoietic cells lack H-2K^b^ expression. Grafted mice received peptide-coated spleen cells on the day of grafting in order to initiate OT-I priming. (**a**) Representative flow cytometry plots (gated on viable CD45.1^+^CD8^+^Vα2^+^ lymphocytes). Upper panel shows lack of division and FucciG expression in quiescent OT-I in LN of a mouse that was neither grafted nor primed. Lower three panels show reponses in a bipolar grafted and primed mouse: draining renal LN, KbKO.βOVA and KbKO.βOVA.βKb grafts. Divided cells in grafted mice were divided into two sectors with the highly divided cells falling into the sector in which CTV was diluted beyond the limit of detection. (**b**) Total divided FucciOT-I in KbKO.βOVA and KbKO.βOVA.βKb bipolar grafts, *P* values calculated by two-tailed ratio paired *t*-test. (**c**) %highly divided and (**d**) % FucciG^+^ OT-I in draining renal LN and grafts of bipolar grafted mice. *P* values were calculated by two-tailed paired *t*-test. Results for individual mice are connected by dashed lines, *n*=6 recipient mice pooled from two independent experiments.

We next took advantage of the FucciOT-I model to assess the role of another potential local driver of T-cell proliferation in the graft, host-derived APC such as inflammatory DC. In order to eliminate any contribution of host APC we first derived a KbKO background. As KbKO mice rejected OT-I cells, we created KbKO BM into B6 chimaeras to use as hosts; thus the hematopoietic cells lacked H-2Kb but were permissive of OT-I cells. These chimaeras were given FucciOT-I T cells and bipolar grafts of KbKO.βOVA islets (lacking H-2K^b^ expression and thus the ability to present OVA antigen) and KbKO.βOVA.βKb islets (both H-2K^b^ and OVA antigen was limited to β cells). As the chimaera lacked H-2K^b^ positive APC it was necessary to administer OVA_257-264_ peptide-coated spleen cells in order to initiate the OT-I response. Success of this priming was shown by CTV-dilution in FucciOT-I cells in the renal LN, although at the time of harvest these were FucciG^−^ and did not appear to be actively dividing (0.1±0.0% FucciG^+^, Fig. 6a,d). Very few OT-I infiltrated the KbKO.βOVA grafts but were clearly expanded within KbKO.βOVA.βKb grafts (RRR of 61.4±26.6 for KbKO.βOVA.βKb: KbKO.βOVA, Fig. 6b, *P*=0.0008, two-tailed ratio paired *t*-test). Thus, even in the absence of cognate antigen on host APC, cognate antigen on parenchymal β cells was sufficient to drive CD8 T-cell expansion at the graft site. The few FucciOT-I T cells present in KbKO.βOVA grafts resembled those in the LN both in terms of their inefficient progression to the highly divided fraction (Fig. 6a,c, 7.5±0.9% highly divided in LN and 19.3±3.3% highly divided in KbKO.βOVA grafts) and lack of FucciG expression (Fig. 6a,d, 0.1±0.0% in LN and 0.1±0.1% in KbKO.βOVA grafts) suggesting that little OT-I proliferation occurred at the graft site in the absence of cognate antigen. By contrast, the OT-I within KbKO.βOVA.βKb grafts were predominantly highly divided (92.3±1.0%, Fig. 6a,c) and some of these were FucciG^+^ and thus actively proliferating (Fig. 6a,d, 5.0±1.2% FucciG^+^). Hence, cognate antigen presentation by islet parenchymal β cells was able to drive *de novo* proliferation of FucciOT-I *in situ*.

### IL-2 is more important at the site of inflammation

**Figure 7:**
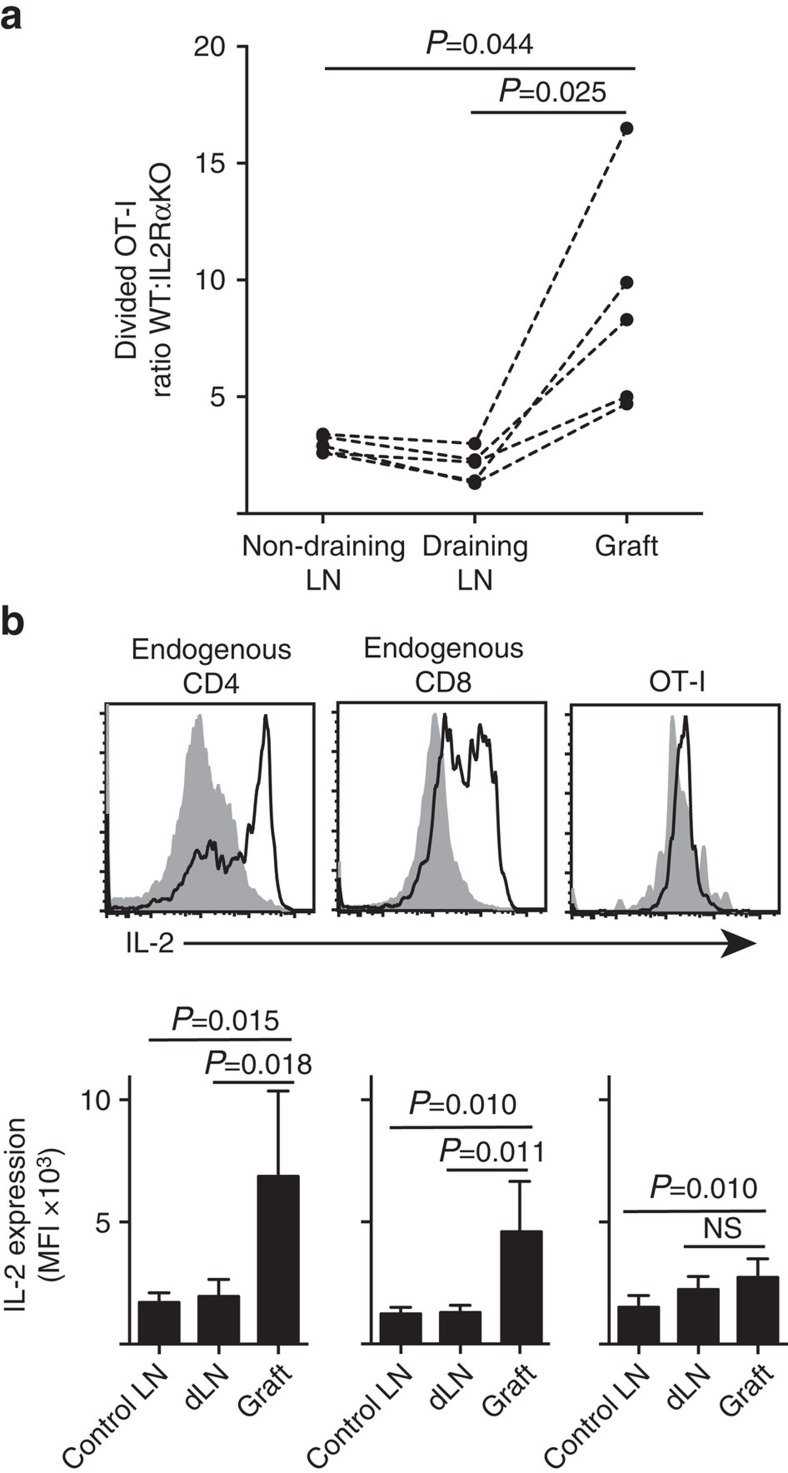
IL-2 is more important for CD8^+^ T-cell expansion at the site of inflammation. (**a**) Ratio of divided IL-2Rα WT:IL-2Rα KO OT-I cells recovered from B6.βOVA islet graft, draining renal LN and non-draining inguinal LN after co-transfer (10^6^ of each) into B6.CD45.1 host mice. Ratios were calculated for individual organs with results for individual mice connected by dashed lines and compared by two-tailed paired *t*-test. Results shown for *n*=5 recipient mice and representative of three independent experiments. (**b**) IL-2 expression in endogenous CD4^+^ and CD8^+^ cells and transferred OT-I cells in B6 recipients of B6.βOVA islet grafts. Upper panels show representative flow cytometry plots for graft draining renal LN (solid grey) and graft (black line). Lower panels summarize mean fluorescent intensity (MFI) of IL-2 expression for renal LNs taken from ungrafted (Control LN), as well as graft and draining renal LN (dLN) of grafted mice. Results shown as mean+s.d., *n*=6 pooled from two independent experiments and compared by two-tailed unpaired *t*-test with Welch's correction.

We show that IL-2Rα expression was more important for CD8^+^ T-cell proliferation and accumulation within peripheral parenchyma (islet grafts) compared to within the site of priming in draining LN (Fig. 7a). In addition, we analysed the response in a second site distant from the site of priming, that is, non-draining LN. CTV-labelled CD8^+^ T cells from OT-I.IL-2RαKO (IL-2Rα^−^CD45.2^+^) and OT-I/Ly5.1 (IL-2Rα^+^, CD45.1^+^CD45.2^+^) mice were co-transferred into B6.CD45.1 host mice (CD45.1^+^) before receiving a single graft of B6.βOVA islets. The distinct CD45 allelic signatures of each of the transferred T-cell populations (in combination with staining for CD8 and Vα2) enabled us to quantify and compare the ratio of divided WT:IL-2RαKO OT-I (Fig. 7a, Supplementary Fig. 3). IL-2Rα WT clearly outcompeted IL-2Rα KO OT-I at the graft site compared to either draining (*P*=0.025, two-tailed paired *t*-test) or non-draining (*P*=0.044, two-tailed paired *t*-test) LN. This is consistent with increased dependence of CD8^+^ T cells on IL-2 at the time of secondary encounter with antigen at the site of inflammation compared to during initial priming in the draining LN.

The heightened dependence on IL-2 for CD8^+^ T-cell proliferation at the site of inflammation inferred a requirement for local IL-2 production. Hence, we examined the potential of various T cells (endogenous host-derived CD4^+^ and CD8^+^ as well as transferred OT-I CD8^+^ T cells) for the capacity to produce IL-2 (Fig. 7b). Cell suspensions prepared from grafts or LN were briefly (4 h) restimulated with PMA and ionomycin before staining for expression of intracellular IL-2. Examination of endogenous host derived CD4^+^ and CD8^+^ T cells provided compelling evidence of IL-2 production at the site of graft inflammation with a clear increase in MFI of IL-2 staining by comparison to either the draining or non-draining LN. IL-2 levels in OT-I CD8^+^ T cells at the site of inflammation were moderately increased over those in the non-draining LN, but did not differ significantly from those in the draining LN. One interpretation of these data is that cognate interaction of OT-I with parenchymal cells induces rapid proliferation but poor IL-2 production, such that the OT-I are dependent on paracrine IL-2 derived from endogenous CD4^+^ and CD8^+^ T cells in the local graft environment. However, an additional experiment indicated that exogenous IL-2 in the absence of parenchymal antigen could not drive OT-I CD8^+^ cell expansion. IL-2 (recombinant hIL-2, 25,000 IU) was administered intraperitoneally to B6 mice that received bipolar grafts of B6.βOVA and B6 islets. Comparison of OT-I recovery in organs recovered from IL-2 or vehicle treated mice at day 6 after grafting showed no significant increase in accumulation of divided OT-I cells in two sites that lack parenchymal antigen namely B6 islet grafts (3±3 in vehicle treated compared to 49±64 in IL-2 treated mice, *P*=0.342) and non-draining inguinal LN (1,648+633 in vehicle treated compared to 1,660+1,023 in IL-2 treated mice, *P*=0.987, mean±s.d., *n*=3, two-tailed unpaired *t*-test with Welch's correction).

## Discussion

Cognate interactions between parenchymal cells (for example, virally infected cells and allografts) as targets and CD8^+^ T cells have long been known. Here we show that parenchymal cells can act as APC *in situ* to drive further expansion of T cells beyond priming in lymphoid tissues. The bipolar graft model provides several advantages to explore the role of APC in T-cell expansion at the site of inflammation. Previous studies relied on comparisons between mice that were manipulated with the intent of altering local antigen presentation. It was not possible to exclude that such manipulations might affect T-cell expansion within draining lymphoid tissue, such that altered local responses might be secondary to an altered pool of T cells primed for infiltration. This is particularly pertinent as ongoing recruitment of T cells occurs during inflammation. The bipolar graft model ensures a common pool of T cells is available for infiltration, thus, enabling a more accurate comparison of local T-cell responses. Furthermore, grafted tissue is amenable to iatrogenic (for example, IRL elimination from CD11c.DTR.GFP.βOVA islets) or genetic (for example, KO of Kb from various islet cells) manipulation of various cells that may contribute to the local response. We showed that the presence of cognate antigen on islet parenchymal β cells but not IRL enhanced the expansion of CD8^+^ T cells at the site of inflammation.

There had been several reports of antigen presentation at sites of inflammation (beyond the initial priming in lymphoid organs) promoting T-cell expansion. Most studies had suggested DC as the APC in these interactions as drivers of local T-cell recruitment, proliferation and survival^12^,^13^,^15^,^16^,^28^. Other studies suggested that non-hematopoietic APC can also contribute to local T-cell expansion. Cognate interactions with endothelial cells have been implicated in recruitment of antigen-specific T cells into peripheral tissues^10^,^11^. A role for parenchymal cells in driving the local T-cell proliferation has also been opined^14^,^29^ but never directly shown. For example, in one study, the proliferation of CD8^+^ T cells specific for pancreatic parenchymal β cell antigen was observed within the pancreas and decreased when MHC expression on β cells was reduced^14^. However, DC were not excluded as the drivers of this response. In another study, selective transgenic expression of OVA antigen in kidney podocytes^29^ led to OT-I proliferation in lymphoid organs in bm-1 reconstituted irradiation chimaeras. Unfortunately, proliferation within the kidney parenchyma was not reported. Our study confirms the importance of cognate antigen on parenchymal cells as a potent driver of local CD8^+^ T-cell proliferation.

It should be noted that our finding that parenchymal cells can reduce T-cell expansion *in situ* does not exclude that other cells (for example, stromal cells, DC) can also play a role. Indeed, we found that there was also moderate T-cell expansion in KbKO.βOVA islets (which completely lacked Kb expression) but only when host hematopoietic cells expressed H-2K^b^. One possible explanation of this finding was that the non-parenchymal APC was a host inflammatory DC that repopulated the islets. A possible role for non-parenchymal APC was also suggested by our examination of IL-2 dependence and IL-2 production by T cells infiltrating B6.βOVA grafts. In this circumstance OT-I can form cognate interactions with parenchymal cells, proliferate rapidly and are highly IL-2 dependent, but appear to be poor producers of IL-2 when compared to endogenous CD4^+^ and CD8^+^ T cells. Given that antigen presentation to CD4^+^ T cells is likely to be dependent on class II expressing cells such as inflammatory DC, it is feasible that T-cell interactions with DC at the site of inflammation are required to provide paracrine IL-2 to CD8^+^ T cells proliferating in response to parenchymal antigen presentation.

There are many implications to cognate parenchyma-induced T-cell expansion. One could speculate that this would selectively enhance cognate responses in target tissues. Hence it would amplify the defence against infection *in situ*. Indeed, it may help to explain the preferential accumulation of cognate T cells over bystander T cells in target tissues^30^. Increased MHC I expression and CD8^+^ T-cell accumulation is a characteristic of human and NOD autoimmune diabetes^31^,^32^. Conversely reduction of MHC levels on target tissues can reduce autoimmune disease^33^; this had been assumed to be due to reduction in target susceptibility, but now could be extrapolated by our findings to be possibly due to reduced T-cell expansion in target tissues.

Our studies add support for a paradigm shift in which the T-cell response is not simply determined by priming events that occur within draining lymphoid tissue. Rather, signals received by the T cell at the site of inflammation are additional critical determinants of the quality and quantity of the local response^34^. The milieu in parenchymal tissues would differ from that of lymphoid organs. As such it would be anticipated that the requirements and characteristics may differ between the classical primary lymphoid response and this ‘mezzanine' response (neither classical primary nor classical secondary responses, which usually takes weeks after a primary response, not a further 3 days). Indeed, here we showed that the primary response in the draining LN is less dependent on IL-2R than the response in peripheral tissues. This finding is consistent with earlier reports that late T-cell proliferation becomes more IL-2 dependent^27^,^35^ particularly in non-lymphoid tissues^36^,^37^. We further define the distinct nature of the antigen presentation requirements of this mezzanine T-cell response. Whereas naïve T cells require cognate antigen presentation on professional APC for their priming in lymphoid organs, ‘mezzanine' T cells are able to respond to cognate antigen on non-professional parenchymal APC. Costimulatory requirements are reduced following T-cell activation (reviewed in ref. 38). Indeed, we recently reported that T-cell costimulation through CD27 and CD28 was principally required before the first division^27^. Hence, it is not surprising that islet parenchymal cells lacking costimulatory molecules^39^,^40^ can nevertheless drive proliferation of T cells that have been recently activated in the LN.

It is well known that interventions that effectively prevent the initiation of immune responses are frequently inefficient if commenced after priming has taken place. The distinct signalling requirements of the mezzanine response further elucidate this observation and also highlight that a more comprehensive understanding of interactions at the site of inflammation may point to the most effective interventions for enhancement or blockade of tissue inflammation.

## Methods

### Mice

B6.Cg-Tg(FucciG1)#639BSi (FucciRed) and B6.Cg-Tg(FucciS/G2/M)#492BSi (FucciGreen) mice were obtained from the Riken BioResource Centre (Hirosawa, Wako, Saitama, Japan)^25^ and crossed to obtain FucciRG mice. B6.129S4-*Il2ra*^tm/Dw^/J mice were obtained from the Jackson Laboratory. OT-I mice^41^ were bred with B6.129S4-*Il2ra*^tm/Dw^/J mice to obtain OT-I.IL-2RαKO mice. An F1 cross between OT-I TCR transgenic mice and B6.SJL-*Ptprca* mice (abbreviated B6.CD45.1) was used to produce OT-I/CD45.1 mice that express the OT-I TCR and both CD45.1 and CD45.2 proteins. C57BL/6-H-2Kb<tm1> mice^42^ that are H-2K^b^ deficient (abbreviated KbKO) were obtained through the NIAID Exchange Program, NIH: 004216. KbKO, C57BL/6 (abbreviated B6), B6.C-*H2-K*^*bm1*^ (abbreviated bm1), B6.RIP-Kb mice^24^ that express the class I H-2K^b^ molecule in β cells under control of the Rat Insulin Promotor (abbreviated βKb) and B6.RIP-mOVA mice^43^ that express OVA in islet β cells under control of the Rat Insulin Promotor (abbreviated B6.βOVA) were crossed to obtain the genotypes described in Supplementary Table 1. CD11c.DTR.GFP (ref. 8) mice were crossed to B6.βOVA mice to obtain CD11c.DTR.GFP.βOVA mice whose DC express the DT receptor and a GFP reporter under control of the CD11c promoter and islet β cells express OVA. Mice were bred and maintained at the Walter and Eliza Hall Institute under SPF conditions. Host mice were male mice at 6–12 weeks old and islet donors were males and females from 6 to 30 weeks old that were sex and age matched within experiments. Blinding was not used. Experiments were performed under approval of the Walter and Eliza Hall Institute Animal Ethics Committee.

### Islet grafts

Islets were isolated by Collagenase P digestion and Histopaque-1077 density gradient centrifugation^44^ then hand-picked and counted. Islets were transplanted under the kidney capsule of host mice that had already received intravenous OT-I T cells. In the case of bipolar graft experiments, the location (upper or lower pole) was randomized between some experiments in order to eliminate graft location as a significant factor in determining the magnitude of the CD8^+^ T-cell proliferative response.

### Depletion of IRL

To deplete IRL from islets, CD11c.DTR.GFP mice were treated intraperitoneally with 5 ng g^−1^ diphtheria toxin (CSL, Parkville, VIC, Australia) either as a single dose on the day before islet recovery or two doses one and two days before islet recovery.

### Preparation of OT-I T cells and adoptive transfer

CD8^+^ T cells were enriched from pooled LN (peripheral and intra-abdominal) and spleen of mice by negative selection: single cell suspensions (approximately 10^8^ cells per ml) were incubated for 30 min with a cocktail of purified monoclonal antibodies containing 20 μg ml^−1^ each of anti-Mac-1 (clone M1/70), anti-F4/80 (clone F4/80), anti-erythrocyte (clone TER-119), anti-GR-1 (clone RB68C5), and anti-class II (clone M5/114) and 100 μg ml^−1^ anti-CD4 (clone GK1.5). Antibodies were obtained from the Walter and Eliza Hall Institute antibody facility. Antibody-coated cells were removed by incubation with goat anti-rat IgG-coupled magnetic beads. In cotransfer experiments CD8^+^ T cells were isolated by the Mouse CD8α+ T-cell isolation kit II (Miltenyi). Negatively selected T cells were generally more than 90% OT-I positive (CD8^+^Va2^+^). Cells were dye-labelled with 5 μM CFSE or CellTrace Violet (Invitrogen). OT-I were administered intravenously to host mice generally on the day before grafting, but up to 4 days before grafting in some of the experiments summarized in Fig. 1. Host mice received 3 × 10^6^ OT-I/CD45.1 or 2 × 10^6^ FucciOT-I. Equal numbers, 10^6^ each, of OT-I/CD45.1 and OT-I.IL-2RαKO were administered in cotransfer experiments.

### FACS analysis

Tissues were digested to single cells with frequent mixing at room temperature in 1 mg ml^−1^ Type III Collagenase (Worthington) and 0.01% w/v Grade II bovine pancreatic DNAse I (Roche) for 30 min with addition of EDTA at a final concentration of 8 mM for the final 5 min. Viable cells were identified as Propidium Iodide negative. Cells were quantified by spiking samples with a known number of fluorescent Calibrite beads. Antibody conjugates were purchased from BD Biosciences unless indicated otherwise. Adoptively transferred OT-I in host mice with or without islet grafts were identified by expression of CD8 (clone 53-6.7, 1:200) and TCR Vα2 (clone B20.1, 1:200) and expression of CD45.1 (Clone A20, 1:150) and CD45.2 (Clone 104, 1:150) proteins that differed from host mice. In most experiments, tissues obtained from mice were immediately digested for FACS analysis. In some experiments, grafts were dissected away from the kidney and cultured for 1 day at 37 °C/10% CO_2_ before digestion for FACS analysis. Graft cultures in six-well plates containing 1 ml of RPMI supplemented with 10% fetal bovine serum were performed by placing grafts, kidney capsule side up, on Millipore Durapore membrane filters resting on a gelfoam platform. IRL within fresh ungrafted islets were identified by expression of CD45 (clone 30-F11, 1:100), CD11c (clone HL3, 1:200) and a GFP reporter transgene. In some experiments IRL were also stained for expression of F4/80 (clone BM8.1, Caltag, 1:100) and MHC H-2K^b^ (clone AF6-88.5, 1:50). β cells within fresh ungrafted islets were identified by high autofluorescence and failure to express either CD45 (clone 30-F11, 1:150) or CD31 (clone MEC13.3, 1:200). MHC H-2K^b^ (clone AF6-88.5, 1:50) expression on β cells was evaluated subsequent to anti-CD3 mAb treatment of islet donor mice (Clone 2C11, WEHI monoclonal antibody facility, 10 μg i.p.). Such anti-CD3 treatment of donor mice was performed in order to upregulate class I expression on β cells as class I levels are very low and difficult to detect in the absence of induction.

### Detection of intracellular IL-2

Cell suspensions prepared from grafts or renal LN were incubated in round-bottom 96-well plates in 200 μl RPMI containing 10% FCS plus 20 ng ml^−1^ PMA and 1 μg ml^−1^ Ionomycin at 37 °C/5% CO_2_. After 1 h BD GolgiStop (0.7 μl ml^−1^) was added, then cells were cultured for an additional 3 h. The cell surface was stained for expression of CD4 (clone RM4-5, 1:400), CD8 (clone 53-6.7, 1:200), CD45.1 (clone A20^−^, eBioscience, 1:100) and CD45.2 (Clone 104, BioLegend, 1:150) enabling identification of host cells (CD4^+^CD8^−^CD45.2^+^ or CD4^−^CD8^+^CD45.2^+^) and adoptively transferred OT-I cells (CD4^−^CD8^+^CD45.1^+^). Cells were then fixed and permeabilized using BD Cytofix/Cytoperm reagents before staining for IL-2 expression (clone JES6-5H4, 1:200) and analysis by flow cytometry.

### Chimaeras

Mice were irradiated with two doses of 5.5 Gy delivered 3 h apart before i.v. reconstitution with 2–8 × 10^6^ BM cells. In the case of mice that received KbKO BM, radioresistant T cells were depleted by intraperitoneal administration of 0.1 ml of T24 (anti-Thy1) culture supernatant on the day following BM.

### Priming with peptide-coated spleen cells

B6 spleen cells at 10^8^ cells per ml were incubated with 2 μg ml^−1^ OVA_257-264_ peptide and 1 μg ml^−1^ LPS (*Escherichia coli* 0111:B4, Sigma), then washed before intravenous injection of 2 × 10^7^ cells into host mice.

### Statistical analysis

Statistical analysis was performed using GraphPad Prism Version 6.0. Responses were compared by two-tailed *t*-tests: Welch's unequal variances test, paired *t*-test or ratio paired *t*-tests as indicated in figure legends. A sample size of at least 3 was based on power analysis of graft data presented in Fig. 1. The relative OT-I expansion between bipolar grafts was derived by calculating the ratio of divided OT-I in grafts placed at opposite poles of individual mice. The ratios obtained for individual mice were then pooled and used to calculate an RRR expressed as mean±s.e.m.

### Data availability

The data that support the findings of this study are available from the corresponding author on reasonable request.

## Additional information

**How to cite this article:** Sutherland, R. M. *et al*. Cognate antigen engagement on parenchymal cells stimulates CD8+ T cell proliferation *in situ*. *Nat. Commun.* **8,** 14809 doi: 10.1038/ncomms14809 (2017).

**Publisher's note:** Springer Nature remains neutral with regard to jurisdictional claims in published maps and institutional affiliations.

## Supplementary Material

Supplementary Information Supplementary Figures, Supplementary Notes and Supplementary References.

Peer Review File
